# Editorial: Research advances and challenges in emerging and re-emerging viral diseases

**DOI:** 10.3389/fmicb.2025.1767138

**Published:** 2026-01-14

**Authors:** Angela Stufano, Gianvito Lanave, George William Carnell, Claudia Maria Trombetta

**Affiliations:** 1 Department of Medical and Surgical Sciences, University of Foggia, Foggia, Italy; 2 Department of Veterinary Medicine, University of Bari Aldo Moro, Valenzano, Bari, Italy; 3 Wolfson Centre for Global Virus Research, School of Veterinary Medicine and Science, University of Nottingham, Nottingham, United Kingdom; 4 Department of Molecular and Developmental Medicine, University of Siena, Siena, Italy

**Keywords:** emerging vector-borne diseases, emerging viral disease, human papillomavirus (HPV), SARS-CoV-2, spillover, viral diagnosis and antiviral agents, zoonosis

Emerging and re-emerging viral diseases continue to pose substantial challenges to public health, veterinary medicine, and global biosurveillance systems. The studies collected in this Research Topic highlight the breadth of current research efforts across virology, immunology, molecular diagnostics, and epidemiology. While spanning distinct pathogens and research methodologies, these contributions converge on common themes: the indispensable role of genomic surveillance, the need for improved vaccines and therapeutics, the importance of understanding host–virus interactions, and the persistent threat of zoonotic and vector-borne viruses.

## Innovations in vaccines, antivirals, and host-directed therapies

A core subset of studies focuses on strategies to counteract viral infections through improved vaccines, antiviral drugs, and immunomodulatory approaches. These contributions emphasize both pathogen-directed interventions and host-targeted strategies that modulate immune responses.

Efforts to refine vaccination against Porcine Reproductive and Respiratory Syndrome Virus (PRRSV) reflect the ongoing challenge posed by rapidly evolving RNA viruses. Liu et al. compared three commercial modified-live PRRSV vaccines in piglets challenged with a heterologous NADC34-like strain, showing that the VR2332-based vaccine provided superior clinical protection relative to two R98-derived vaccines, reducing clinical signs, viral loads, and tissue damage, although none achieved sterilizing immunity.

Complementing this work, Tu et al. explored the adjuvant potential of a botanical extract combination derived from *Curcuma zedoaria* and *Astragalus membranaceus*, demonstrating enhanced antibody responses, lymphocyte proliferation, and cytokine modulation when used with an inactivated PRRSV vaccine, with inhibition of viral replication *in vitro* and improved growth performance *in vivo*.

Meanwhile, in the context of human respiratory viruses, Cai et al. performed a large multicenter comparison of *Baloxavir marboxil* and *Oseltamivir* in patients with influenza A, demonstrating that *Baloxavir* produced faster symptom resolution when administered early, with no increase in adverse events and consistent real-world effectiveness.

Host-directed strategies are represented by Sun et al., who evaluated the regulatory role of RIOK3 in respiratory syncytial virus (RSV) infection. RIOK3 deletion impaired type I interferon signaling, increased viral replication, and altered macrophage inflammatory responses, highlighting RIOK3 as a potential therapeutic target for modulating innate immunity and reducing immunopathology.

Finally, Coppola et al. provide an innovative perspective on synergistic immune protection in lentiviral infection, demonstrating that combined use of Rhesus cytomegalovirus–vectored SIV vaccines and neutralizing antibodies enhances viral control following challenge, beyond the effects of either modality alone.

## SARS-CoV-2: mechanisms, evolution, and clinical management

Several contributions address SARS-CoV-2 from molecular, computational, clinical, and methodological perspectives, underscoring the continued need for multidisciplinary research amid ongoing viral endemicity.

Zeng et al. report a compelling case series of two very elderly patients co-infected with hepatitis C virus (HCV) and SARS-CoV-2 who were successfully treated with sequential antiviral therapy—sofosbuvir/velpatasvir followed by nirmatrelvir/ritonavir—resulting in complete clearance of both viruses without adverse reactions, and sustained HCV remission after 9 months.

Host nutritional status and pathogenesis are examined by Carolin et al., who assessed the impact of iron deficiency or overload on SARS-CoV-2 Omicron XBB infection in mice. Although dietary iron imbalance produced modest inflammatory transcriptional changes, it did not significantly affect viral load or lung pathology, indicating that pre-existing iron status is not a primary driver of acute COVID-19 severity in this model.

A future-oriented perspective is offered by Liu et al., who developed DARSEP, a deep learning–based model integrating reinforcement learning to predict spike protein evolution. By simulating mutational trajectories and scoring predicted viral fitness, DARSEP identifies variants with increased immune escape potential and highlights likely future evolutionary hotspots, supporting proactive vaccine design and surveillance efforts.

Immune differences across age groups are addressed by Chen et al., whose case–control study of severe COVID-19 revealed that adults exhibit marked lymphopenia and heightened pro-inflammatory cytokines—IL-1β, IL-6, IL-8, IFN-γ, TNF-α–whereas pediatric patients show more balanced inflammatory profiles, correlating with reduced disease severity.

Finally, Dong et al. introduce a highly sensitive intracellular SARS-CoV-2 main protease reporter system (ICMP), capable of detecting protease activity from 13 coronaviruses and enabling antiviral screening without high-containment facilities. The platform demonstrates a >1,000-fold signal increase upon protease activity and reliably detects low-MOI infection, making it a powerful tool for drug discovery and coronavirus surveillance.

## Human papillomavirus: genotype dynamics, vaccination impact, and oncogene function

Studies dedicated to human papillomavirus (HPV) offer complementary insights into the epidemiology of high-risk HPV genotypes and the impact of screening and vaccination strategies at the population level.

Using data from more than 18,000 screening events, Wei et al. analyzed HPV prevalence and genotype distribution among women in Shenzhen, China, reporting an overall prevalence of 12.39% with bimodal age peaks in young and older women. High-risk genotypes HPV52, HPV58, and HPV16 predominated, and hr-HPV positivity was strongly associated with cytologic and histopathologic abnormalities, especially in CIN II/III lesions. Importantly, age-stratified analysis revealed declines in vaccine-preventable genotypes among women under 30 following the rapid uptake of nonavalent vaccination since 2022.

At the molecular level, Cao et al. investigated the functional impact of the P16L variant of HPV16 E5, showing that the substitution enhances proliferation, migration, and colony formation while inhibiting apoptosis in epithelial cells. Variants influenced the expression of Cyclin D1 and BAX, suggesting that naturally occurring oncoprotein polymorphisms may modulate oncogenic potential and contribute to clinical heterogeneity.

Together, these studies highlight both the population-level success of vaccination programs and the importance of understanding viral genetic variation that may influence pathogenicity.

## Emerging and re-emerging viruses: ecology, evolution, and molecular surveillance

The Research Topic brings together a diverse body of research examining emerging pathogens across human, veterinary, and vector-borne systems, collectively underscoring the essential role of integrated genomic, ecological, and epidemiological surveillance.

Within this context, Salem et al. offer a conceptual contribution that elucidates the phenomenon of immune imprinting across orthoflaviviruses. Their analysis demonstrates how original antigenic sin can shape the hierarchy of memory B-cell responses following sequential flavivirus exposures, at times producing cross-neutralizing protection but in other circumstances favoring non-neutralizing antibody profiles capable of driving antibody-dependent enhancement, particularly in dengue. The immunological model presented illustrates the delicate balance between immune memory and viral escape and highlights the need for vaccines designed to elicit broad, cross-reactive immunity across diverse antigenic landscapes.

A complementary example of viral emergence in the veterinary context is provided by Goraichuk et al., who document the first detection of avian metapneumovirus subtype A (aMPV-A) in the United States. By sequencing complete and near-complete viral genomes, the authors identify previously unreported mutations in the G glycoprotein, a key determinant of receptor binding and tissue tropism. Their validation of a refined RT-qPCR assay capable of differentiating viral subtypes with high sensitivity enhances the diagnostic capacity necessary for early detection, enabling better control measures within poultry populations and refining regional surveillance strategies.

In a related examination of zoonotic potential, Markarian and Abrahamyan synthesize recent advances in Sosuga virus research, emphasizing progress in understanding its replication dynamics and pathogenic determinants. Given the virus's origin in bat reservoirs and its phylogenetic affinity to highly pathogenic paramyxoviruses such as Nipah and Hendra viruses, the authors argue for the urgent expansion of One Health–based surveillance frameworks to better characterize spillover risks and improve preparedness for potential future outbreaks.

Emerging viral diversity is also evident in the study by Hu et al., who report the presence of Severe Fever with Thrombocytopenia Syndrome Virus (SFTSV) in Jiangxi Province and identify segmental reassortment events among genotypes C4, C5, and J1. Although viral RNA was not detected in animal or tick samples collected around confirmed cases, phylogenomic and recombination analyses demonstrated clear evidence of genetic exchange. The identification of reassortant strains in humans but not in sampled vectors or reservoir hosts suggests gaps in ecological sampling and highlights the ongoing need for comprehensive surveillance in regions experiencing newly recognized transmission.

Diagnostic capacity likewise emerges as a central theme in the work of Wang et al., who developed a highly sensitive duplex qPCR assay capable of distinguishing between classical (GI) and variant (GII) subtypes of porcine epidemic diarrhea virus (PEDV). As GII strains now predominate globally and classical GI-based vaccines offer limited cross-protection, the authors' contribution provides an essential tool for guiding both outbreak management and vaccine deployment, allowing for more precise interventions within the swine industry.

The evolutionary plasticity of emerging viruses is further illustrated by Guo et al., who characterize a Getah virus (GETV) variant implicated in severe mortality among piglets. Their genomic analysis identifies distinctive amino acid substitutions in the E2 envelope protein, including the acquisition of an additional glycosylation site and multiple residues undergoing positive selection. Such modifications may influence viral infectivity, immune evasion, or host-range expansion, reinforcing the idea that GETV, although traditionally considered a veterinary pathogen, warrants close monitoring due to its expanding geographic distribution and serological evidence of human exposure.

In the human health domain, Perez et al. analyze a Yellow Fever Virus (YFV) genotype II strain identified through metagenomic surveillance in the Colombian Amazon. Their phylogenetic and phylodynamic analyses reveal that YFV persistence in the region is sustained not by repeated external introductions but by continuous internal circulation, facilitated by selective pressures that have generated unique antigenic signatures.

Finally, Chang et al. offer a long-term epidemiological perspective through their analysis of varicella-zoster virus (VZV) disease burden in China from 1990 to 2021. Their findings demonstrate a steady decline in mortality and disability-adjusted life years in contrast with relatively stable incidence and prevalence rates. Age and gender disparities remain evident, and projections based on ARIMA modeling suggest that although the national burden is likely to decrease modestly by 2030, the combination of an aging population and persisting susceptibility will sustain VZV as a meaningful public health concern.

## Future directions

Across the diverse pathogens represented in this Research Topic, a series of shared priorities becomes evident, each reflecting fundamental challenges in the surveillance and control of viral emergence. Central among these is the need for robust and sustained genomic monitoring. The identification of reassortment events in SFTSV (Hu et al.), the detection of positively selected mutations in GETV (Guo et al.) and YFV (Perez et al.), and the rapid antigenic diversification observed in SARS-CoV-2 (Liu et al.) collectively demonstrate how quickly viral populations can shift in response to ecological pressures, host immunity, or stochastic evolutionary processes.

Equally critical is the adoption of comprehensive One Health approaches. The spillover risks associated with mosquito-borne viruses such as YFV (Perez et al.), tick-borne agents like SFTSV (Hu et al.), bat-associated pathogens including Sosuga Virus (Markarian and Abrahamyan), and livestock-affecting viruses such as GETV (Guo et al.), illustrate how human health is intrinsically linked to animal and environmental systems. The interconnectedness of these domains demands surveillance frameworks that integrate ecological monitoring, veterinary diagnostics, and human epidemiology to anticipate spillover events and mitigate their consequences.

In parallel, the studies in this Research Topic highlight the pressing need for next-generation vaccines and therapeutics that provide broad, durable, and cross-reactive immunity. Insights into immune imprinting, the immunological benefits of adjuvant enhancement, and the promising effects of combined-modality vaccination strategies collectively point to the need for platforms that can respond effectively to highly variable or persistently circulating viruses (Tu et al.; Cai et al.).

Methodological innovation plays an indispensable role in this effort. Advances such as duplex qPCR assays for PEDV (Wang et al.) or intracellular protease reporters for SARS-CoV-2 (Dong et al.) exemplify how improved diagnostic tools can accelerate detection, guide therapeutic development, and strengthen outbreak response capacities.

Finally, the importance of understanding host factors and immunopathogenesis emerges as a recurrent theme. Age-dependent differences in immune responses (Chen et al.), the influence of nutritional status (Carolin et al.), and the regulatory roles of host genetic determinants collectively highlight the complex interplay between viral infection and host biology. Such perspectives are essential for refining clinical management strategies, identifying vulnerable populations, and tailoring interventions to specific immunological contexts.

Taken together, the studies in this Research Topic illustrate the evolving and multifaceted challenges associated with managing viral emergence and re-emergence. As global ecological pressures, climate change, and patterns of human mobility continue to reshape the landscape of infectious diseases, the need for interdisciplinary research and coordinated public health strategies becomes increasingly urgent. The insights provided here underscore not only the progress made but also the substantial work that lies ahead in preparing for the next generation of viral threats.

